# Low-intensity pulsed ultrasound restores mitochondrial dynamics and function in lipopolysaccharide-stimulated astrocytes

**DOI:** 10.48101/ujms.v131.13678

**Published:** 2026-03-09

**Authors:** Umut Kerem Kolac, Aysegul Turkkol, Mahmut Alp Kılıc, Gizem Donmez Yalcın, Abdullah Yalcın, Mehmet Dincer Bılgın

**Affiliations:** a Department of Medical Biology, Faculty of Medicine, Aydin Adnan Menderes University, Aydin, Turkey; b Department of Biophysics, Faculty of Medicine, Aydin Adnan Menderes University, Aydin, Turkey

**Keywords:** LIPUS, lipopolysaccharide, mitochondrial dynamics, astrocyte

## Abstract

**Background:**

Low-intensity pulsed ultrasound (LIPUS) is a non-invasive therapeutic modality with growing potential in the treatment of neurodegenerative diseases. However, its mechanistic role in regulating mitochondrial homeostasis in astrocytes under inflammatory stress remains poorly understood. This study aimed to investigate the effects of LIPUS on mitochondrial dynamics, morphology, oxidative stress, mitochondrial membrane potential, and mitochondrial stress response in an *in vitro* model of neuroinflammation.

**Methods:**

Normal Human Astrocytes (NHA) were stimulated with lipopolysaccharide (LPS; 0.5 µg/mL, 24 h) and subsequently treated with LIPUS (1 MHz, 50% duty cycle, 100 Hz, 15 min) at intensities of 100, 300, or 500 mW/cm^2^. The expression of mitochondrial fusion (MFN1, MFN2, OPA1) and fission (DRP1, FIS1) markers was analyzed using qPCR. Mitochondrial morphology was evaluated by confocal microscopy, while reactive oxygen species (ROS) levels and mitochondrial membrane potential (ΔΨm) were measured using specific fluorescent probes. Expression of mitochondrial stress-related genes (*PGC1α, CLPP, HSP60, LONP1*) was also assessed.

**Results:**

LIPUS treatment, particularly at 300 mW/cm^2^, significantly enhanced the expression of mitochondrial fusion markers while suppressing fission markers in a dose- and time-dependent manner, with peak effects observed 4 h post-treatment. Confocal imaging revealed that LIPUS mitigated LPS-induced mitochondrial fragmentation. Additionally, LIPUS reduced ROS accumulation, preserved ΔΨm, and attenuated the LPS-induced upregulation of mitochondrial stress-related genes, suggesting modulation of both stress response and biogenesis.

**Conclusion:**

LIPUS ameliorates mitochondrial dysfunction in inflamed astrocytes by restoring mitochondrial dynamics and reducing stress signaling, supporting its potential as a therapeutic strategy for neuroinflammation-associated neurodegenerative disorders.

## Introduction

Neurodegenerative diseases are destructive conditions with rapidly increasing prevalence and no definitive cure (1). Studies have shown that neuronal cell death can trigger an inflammatory response and that inflammation alone can lead to neurodegeneration (2). The development and advancement of numerous neurodegenerative diseases, including Alzheimer’s disease, Parkinson’s disease, amyotrophic lateral sclerosis, and frontotemporal dementia, are significantly influenced by neuroinflammation (3). Recent studies have shown that in addition to microglia, astrocytes in the brain also play an active role in initiating and regulating immune responses in the central nervous system (CNS) (4, 5). While astrocytes are traditionally known for their supportive role for neurons, they are also capable of releasing proinflammatory cytokines such as interleukin-6 (IL-6), tumor necrosis factor (TNFα), and interleukin-1β (IL-1β) in response to pathological conditions (6). This highlights the importance of studying the role of astrocytes in neuroinflammation.

Endotoxins known as lipopolysaccharides (LPS) are originated from the outer membrane of gram-negative bacteria, which incite an inflammatory response that results in widespread harm to the body (7). Studies have reported that LPS induces an inflammatory response by promoting the release of various proinflammatory cytokines, including IL-1β, TNFα, IL-6, IL-18, and cyclooxygenase-2 (COX-2) (8, 9). LPS is commonly used to induce inflammation in both *in vivo* and *in vitro* neurodegeneration models (2, 10, 11).

Mitochondria are highly dynamic organelles that undergo continuous changes in shape, size, and location within cells. Mitochondrial dynamics involve two opposing processes: fusion and fission. Fusion enables the formation of large interconnected mitochondrial networks, while fission leads to the production of small individual mitochondria (12). These processes are tightly regulated by various proteins and signaling pathways, including the dynamin-related GTPases, mitofusins (MFN1 and MFN2), and optic atrophy 1 (OPA1) for fusion and dynamin related protein1 (DRP1) and fission protein1 (FIS1) for fission (13). Dysregulation of mitochondrial dynamics has been implicated in various human diseases, including neurodegenerative disorders (14), cancer (15), and metabolic diseases (16).

It has been previously reported that mitochondrial fission is upregulated and mitochondrial fragmentation is triggered in LPS-induced neuroinflammation (17, 18). Evidence shows that the balance of mitochondrial dynamics is disrupted under neuroinflammatory conditions, leading to an increase in the production of reactive oxygen species (ROS) (19), defects in mitochondrial protein folding (20), loss of mitochondrial membrane potential (Δψm) (21), and impaired mitochondrial biogenesis (22).

Ultrasound has been used for diagnostic and therapeutic purposes in medicine for years. Low-intensity pulsed ultrasound (LIPUS) generates pulsed waves with low intensity, making it a distinct type of ultrasound (23). LIPUS maintains the delivery of acoustic energy to the intended tissue while generating minimal thermal impact, as a result of its low intensity and pulsed waveform (24). LIPUS exhibits a range of therapeutic applications, including the acceleration of bone fracture healing in clinical treatments and animal models, be effective in tendon and soft tissue regeneration (25), reduce cerebral edema (26), provide neuromodulation, and inhibit neuroinflammatory responses (27).

The proinflammatory responses affected by LIPUS have been demonstrated in many studies (5, 28–31). However, studies investigating the role of this effect on mitochondrial dynamics and mitochondrial dysfunction are needed. In our study, we showed the effects of LIPUS treatment on mitochondrial dynamics, ROS-positive cell ratios, messenger RNA (mRNA) levels of mitochondrial antioxidant enzymes, mitochondrial membrane potential, and mRNA levels of genes involved in proper mitochondrial protein folding in LPS-stimulated astrocytes.

## Materials and methods

### Cell culture

In the study, Normal Human Astrocytes (NHA; Cat. No: CC-2565, Lonza) cell line was used. NHA cultured using an Astrocyte Growth Medium Bullet Kit (Cat. No: CC-3186, Lonza) in dulbecco’s modified eagle medium (DMEM) containing 10% fetal bovine serum, 4.5% glucose, 2 mM L-glutamine 1% penicillin / streptomycin, and reagents from BulletKits (Lonza). The cells were incubated at 37°C in an atmosphere containing 5% CO_2_. Once the cell density exceeded 70%, fresh culture medium was introduced and the cells were transferred to a new culture vessel. After 24 h of seeding the cells into 6-well plates, 0.5 µg/mL LPS (*E. coli* 0111:B4 strain, Cat. No: tlrl-3pelps, InvivoGen) was applied for 24 h, dissolved in endotoxin-free water. Endotoxin-free water was given to the control cells.

### LIPUS application

LIPUS was produced using a therapeutic ultrasound device (BTL-5710 Sono Simulator) with a probe area of 5 cm^2^, operating at 1 MHz, 50% duty cycle, 100–500 mW/cm^2^ (32, 33), and a repetition frequency of 100 Hz. To prevent thermal effects during the application, an ultrasound transmission medium filled with distilled water was placed between the probe and the cell culture plate at a distance of 8 cm, as previously described (34). LIPUS treatment was performed 24 h after LPS addition, and applied to each well of the 6-well plate for 15 min (5).

### Cell viability assay

To determine cell viability after LIPUS and LPS applications, (3-(4,5-dimethylthiazol-2-yl)-2,5-diphenyltetrazolium bromide) tetrazolium (MTT) assay was performed. The culture medium was removed from the cells and replaced with new medium containing 10% MTT. The cells were incubated at 37°C in a 5% CO_2_ atmosphere for 1.5 h. After the incubation period, the medium containing MTT was discarded, and DMSO in the same volume as the medium was added to each well to dissolve the formazan crystals. The absorbance at 570 nm was measured to calculate cell viability as a percentage of the control group.

### Real-time PCR

After LIPUS and LPS treatments were applied to the cells in 6-well plates, total RNA was isolated using trizol (Cat. No: 301-001, GeneAll), and cDNA synthesis was performed for each sample as previously described (Cat. No: W2211, WizScript) (16). The quantitative polymerase chain reaction (qPCR) reaction mix was prepared by combining the master mix, forward and reverse primers (Table 1), probe, and cDNA sample. qPCR reaction mix was added to the PCR tubes, and the tubes were loaded into the qPCR instrument (CFX96 Touch System, Biorad). qPCR cycling condition were 95°C for 10 min for initial denaturation, followed by 40 cycles of 95°C for 15 sec and 55°C for 60 sec for annealing and extension. For each sample, the CT (cycle threshold) values of both the gene of interest and a reference gene (*β-actin*) were determined. The 2-^ΔΔCT^ method was used to calculate the fold change in gene expression.

**Table 1:** Primer sequences used in qPCR reaction.

Gene	Forward (5′-3′)	Reverse (5′-3′)
*MFN1*	CCTGTTTCTCCACTGAAGCAC	CCTCACCAATGATGGAAAGC
*MFN2*	ACACATGGCTGAGGTGAATG	CGTCCAGAACCTGTTCTTCTG
*OPA1*	GGATTGTGCCTGACATTGTG	AAGGCTTTCAACAATCTTGTCA
*DRP1*	CAGTGTGCCAAAGGCAGTAA	GATGAGTCTCCCGGATTTCA
*FIS1*	CTTGCTGTGTCCAAGTCCAA	GCTGAAGGACGAATCTCAGG
*MnSOD*	AAGGGAGATGTTACAGCCCAGATA	TCCAGAAAATGCTATGATT
*GPX1*	GGGACTACACCCAGATGAA	TCTCTTCGTTCTTGGCGTTC
*CLPP*	GCCAAGCACACCAAACAGA	GGACCAGAACCTTGTCTAAG
*HSP60*	CACCGTAAGCCTTTGGTCAT	CTTGACTGCCACAACCTGAA
*PGC1α*	GACTGGCGTCATTCAGGAG	TCAGGAAGATCTGGGCAAAG
*β-ACTIN*	AACTGGGACGACATGGAGAA	GAAGGTCTCAAACATGATCTGG

### Confocal microscopy and mitochondrial morphology analysis

Mitochondrial morphology was assessed via live-cell confocal imaging to evaluate structural alterations under inflammatory and therapeutic conditions. Astrocytes were seeded at a density of 1 × 10⁵ cells per well into confocal dishes (Ibidi; Cat. No: 81156, Germany) and allowed to adhere overnight. After completion of experimental treatments (LPS and/or LIPUS), cells were gently washed with pre-warmed phosphate-buffered saline (PBS) and incubated in phenol red-free DMEM containing 100 nM MitoTracker™ Red FM (ABP Bioscience, Cat. No: C054) for 30 min at 37°C. The dye stock was dissolved in dimethyl sulfoxide (DMSO) and diluted to the working concentration immediately before use.

Live-cell imaging was performed using a Zeiss laser scanning microscopy (LSM) 900 confocal laser scanning microscope equipped with 10× and 20× objectives. Images were acquired using an excitation/emission wavelength of 580/644 nm, and identical acquisition settings (laser power, gain, exposure time) were used across all experimental groups to ensure comparability. Drift correction was applied during image acquisition, and care was taken to minimize photobleaching and movement artifacts. For each group, images were captured from at least five different randomly selected fields, and ~100 individual cells were analyzed per condition.

Mitochondrial structures were analyzed using the Mitochondria Analyzer plugin in Fiji/ImageJ software (NIH, USA). For quantitative morphometric analysis, 20 representative mitochondria were measured per cell, corresponding to a total of approximately 2,000 individual mitochondria per experimental group. Morphological quantification included aspect ratio (AR) – the ratio of the major to minor axis – and form factor (FF), the reciprocal of circularity, both of which serve as established metrics for mitochondrial elongation and branching. Standardized thresholding and size filtering parameters were applied uniformly across all images to ensure objective and reproducible segmentation of mitochondrial particles. Confocal images used for presentation were processed to maintain consistent contrast and brightness across panels. High-magnification (20×) images were presented in the main figure panel, while low-magnification (10×) overview images were included in the supplementary figure to illustrate cell population-level distribution of mitochondrial networks.

### Determination of ROS

Intracellular ROS levels were assessed using the DCFDA/H_2_ DCFDA Cellular ROS Assay Kit (Abcam, Cat. No. ab113851). Cells were washed with PBS and incubated with 2′,7′-dichlorodihydrofluorescein diacetate (DCFDA) working solution at 37°C for 30 min in the dark. After staining, cells were gently washed with PBS, resuspended in assay buffer, and immediately analyzed by flow cytometry. Flow cytometric analysis was performed using a fluorescein isothiocyanate (FITC) detection channel, and 20,000 events per sample were acquired to ensure robust statistical representation. Forward and side scatter parameters were used to exclude debris and cell aggregates. ROS levels were quantified using a two-gate strategy, in which cells were classified as ROS-negative (DCF-low) or ROS-positive (DCF-high) based on fluorescence intensity thresholds defined from unstained and control samples. Identical gating parameters were applied across all experimental groups to allow direct comparison.

### Determination of mitochondrial membrane potential

The Muse Mitopotential Kit (Cat. No: MCH100110, Luminex) was used to measure Δψm levels. In short, assay buffer was used to resuspend NHA cells after washing them with PBS. The cells were then stained with Muse Mitopotential reagent, which contains a fluorescent probe that accumulates in mitochondria with intact Δψm, for 20 min. at 37°C in the dark. Cells were then treated for 5 min with 7-Aminoactinomycin D (7-ADD), a fluorescent intercalator that indicates cell death. After staining, the cells were analyzed using a Guava Muse Cell Analyzer (Luminex), and the data were analyzed using Muse software. Software provided the percentage of depolarized/polarized cells.

Rhodamine 123 is a type of fluorescent positively charged dye that can accumulate in mitochondria through a process that relies on the membrane potential. After treatment with LIPUS, LPS-stimulated cells washed with PBS and stained with Rhodamine 123 (Invitrogen) at a final concentration of 25 μM for 30 min at 37°C in the dark. After staining, cells were washed three times with PBS to remove excess dye. Fluorescence microscopy was performed using Zeiss axiocam ICc5 camera. Images were acquired using Zen software (Zeiss) and analyzed using ImageJ software (National Institutes of Health). Each experiment was performed in triplicate. Five images were captured from each well, and for the calculation of fluorescence intensity, 50 cells per mm^2^ were used from each image.

For the assessment of early changes in mitochondrial membrane potential, Rhodamine 123–stained cells were additionally analyzed by flow cytometry. Following LPS stimulation and LIPUS treatment, cells were stained with Rhodamine 123 as described above and analyzed using the FITC channel, with 20,000 events acquired per sample. The percentages of polarized and depolarized cells were quantified using an identical gating strategy across all experimental groups.

### Statistical analysis

Statistical analysis was performed using GraphPad Prism 9 software (GraphPad Software, San Diego, CA, USA). The normality of the data was determined using the Shapiro–Wilk test. One-way ANOVA Tukey test was used for comparing normally distributed groups, while Kruskal–Wallis test was used for non-normally distributed groups. Results with *P*-value < 0.05 were considered significant.

## Results

### LIPUS promotes mitochondrial fusion and suppresses fission in astrocytes

**Figure 1:**
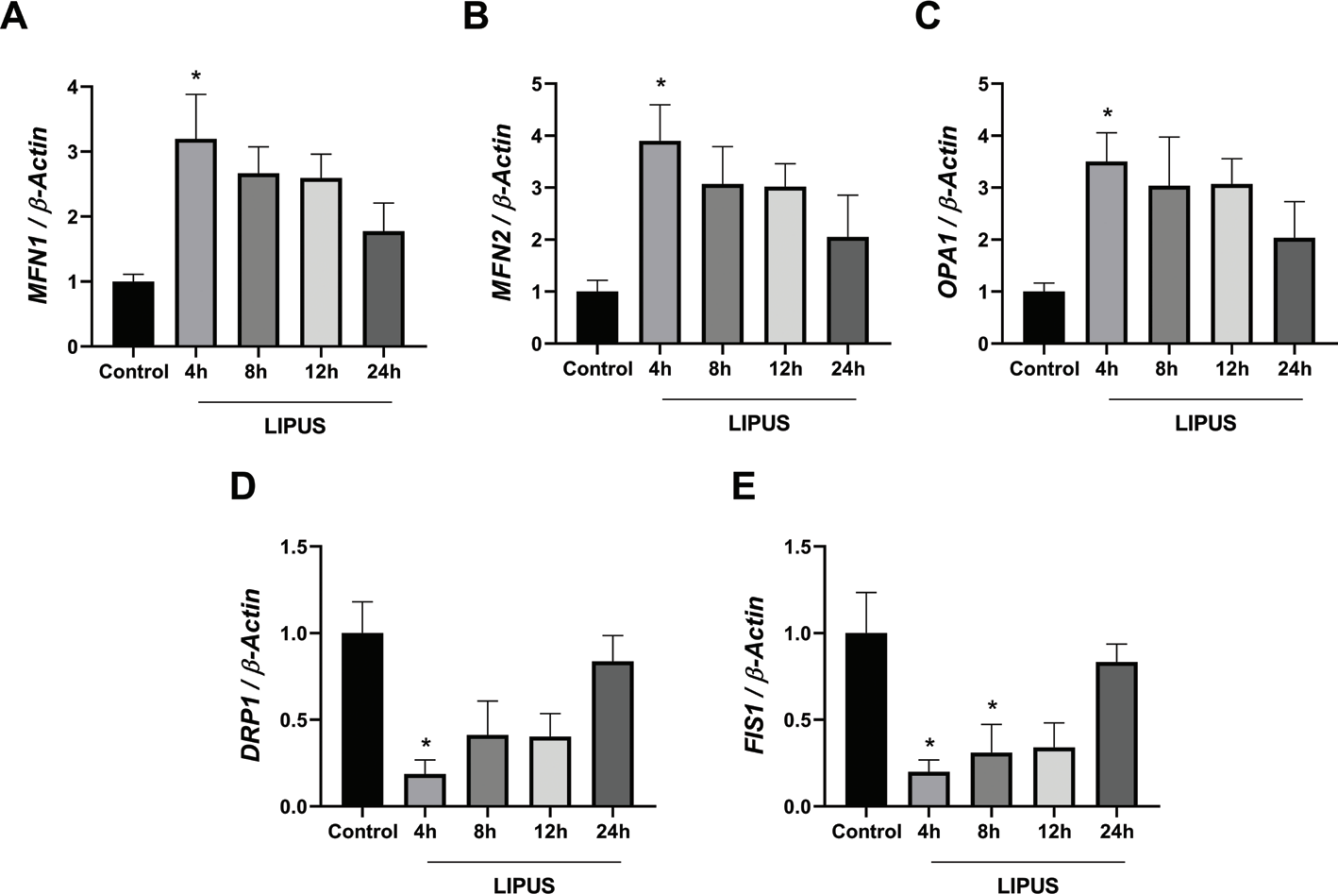
Time dependent effect of LIPUS on mitochondrial fission and fusion genes mRNA levels. mRNA expressions of *MFN1* (A), *MFN2* (B), *OPA1* (C), *DRP1* (D), and *FIS1* (E) at 4, 8, 12, and 24 h following LIPUS treatment (1 MHz, 50% duty cycle, 100 mW/cm^2^, repetition frequency of 100 Hz for 15 min.). Fold changes were normalized to *β-actin* and calculated relative to control. Data are presented as mean ± standart error of the mean (SEM). qPCR reactions were run in triplicates in three independent experiments. *P < 0.05 vs. control. LIPUS: Low-intensity pulsed ultrasound.

Figure 1 depicts the time-dependent changes in mRNA expression levels of genes involved in mitochondrial dynamics following LIPUS treatment at a power intensity of 100 mW/cm^2^. Accordingly, the mRNA expressions of *MFN1* (Figure 1A), *MFN2* (Figure 1B), and *OPA1* (Figure 1C) significantly increased 4 h after LIPUS application. Although their expressions were higher than control cells at 8, 12, and 24 h, they gradually decreased over time. mRNA expressions of mitochondrial fission markers were reduced to the lowest level at 4 h after LIPUS treatment (Figure 1D and E). A significant decrease in *FIS1* mRNA expression was detected even 8 h after LIPUS application (Figure 1E).

To further investigate whether LIPUS initiates early transcriptional regulation of mitochondrial dynamics, fusion and fission gene expression was additionally analyzed at earlier time points. As shown in Supplementary Figure 3, LIPUS treatment induced a progressive upregulation of fusion-related genes as early as 1–2 h, accompanied by a concomitant reduction in fission-related gene expression, particularly *FIS1*. Our results demonstrated that the optimum effect of LIPUS treatment on mitochondrial dynamics occurred at 4 h, and subsequent experiments continued with LIPUS treatment for 4 h.

### LIPUS application downregulates LPS-induced mitochondrial fragmentation

**Figure 2:**
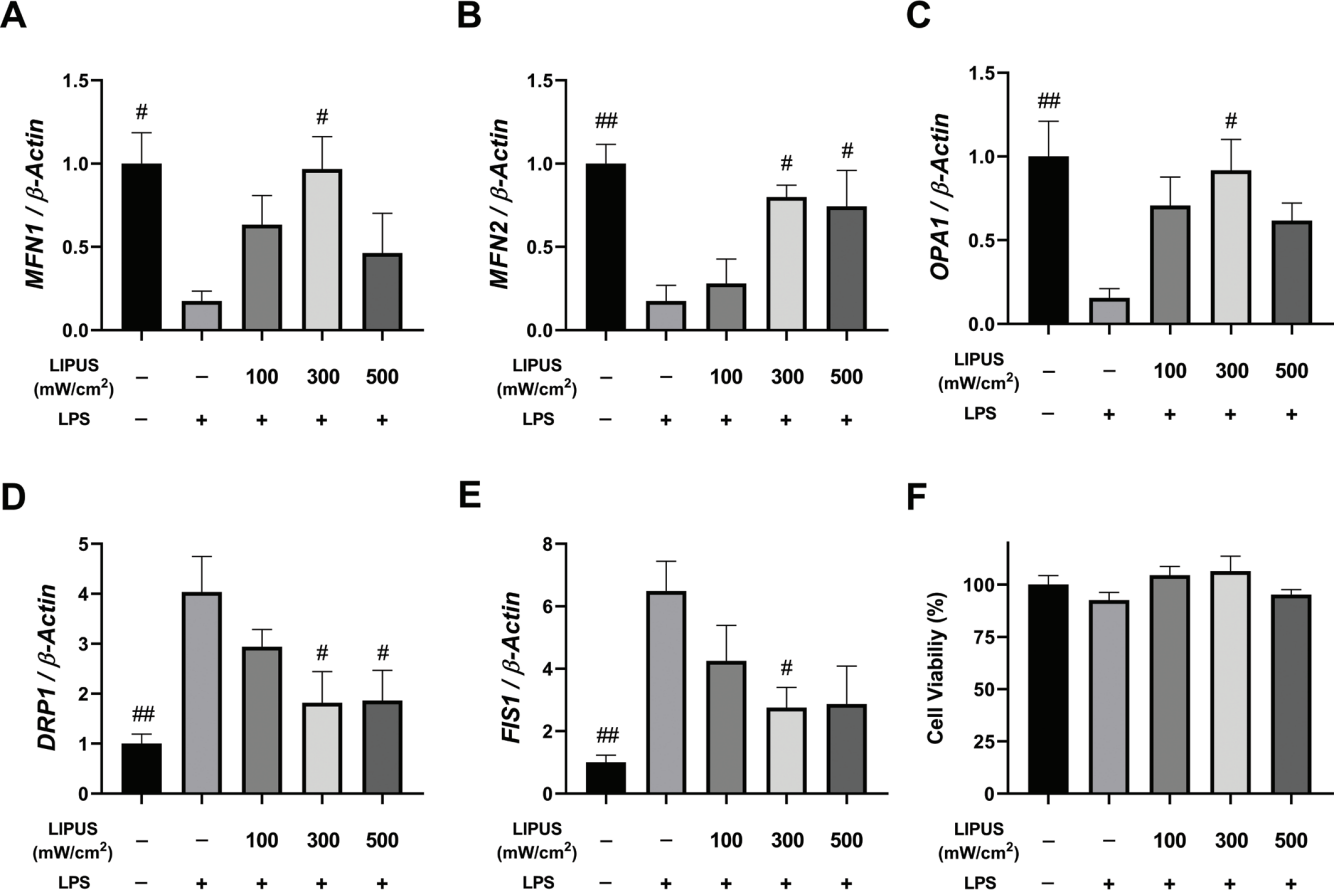
Effects of LIPUS power intensities on mitochondrial fission and fusion genes mRNA levels. mRNA expressions of *MFN1* (A), *MFN2* (B), *OPA1* (C), *DRP1* (D), and *FIS1* (E) in LPS-treated (0.5 µg/mL for 24 h) astrocytes following LIPUS treatment with intensities of 100, 300, and 500 mW/cm^2^ after 4h. Control cells were treated with endotoxin-free water. Fold changes were normalized to *β-actin* and calculated relative to control. Analysis of MTT cell viability (%) relative to control (F). MTT data are presented as mean ± SEM of three independent experiments. qPCR reactions were run in triplicates in three independent experiments. ^##^*P* < 0.01, ^#^*P* < 0.05 vs. LPS. LIPUS: Low-intensity pulsed ultrasound; LPS: lipopolysaccharide.

LPS treatment induces mitochondrial fission and significantly reduces mRNA expression levels of fusion genes in astrocytes. Three different power densities of LIPUS (100, 300, and 500 mW/cm^2^) were applied to cells stimulated with LPS for 24 h, and mRNA expression levels of genes involved in mitochondrial fission and fusion were determined 4 h later. As a result, LIPUS at 300 mW/cm^2^ power intensity significantly increased *MFN1* (Figure 2A) and *OPA1* (Figure 2C) mRNA expressions compared to LPS-treated cells. *MFN2* mRNA expression levels also significantly increased at both 300 mW/cm^2^ and 500 mW/cm^2^ power densities (Figure 2B). In contrast, *DRP1* mRNA expressions showed a significant decrease with LIPUS application (300 and 500 mW/cm^2^) compared to the LPS group (Figure 2D). Similarly, LIPUS applied at a power intensity of 300 mW/cm^2^ reduced *FIS1* expressions (Figure 2E). Although cell viability decreased in LPS-treated cells compared to control cells, this difference was not significant. Co-application of LPS and LIPUS did not affect cell viability compared to the control group (Figure 2F).

**Figure 3:**
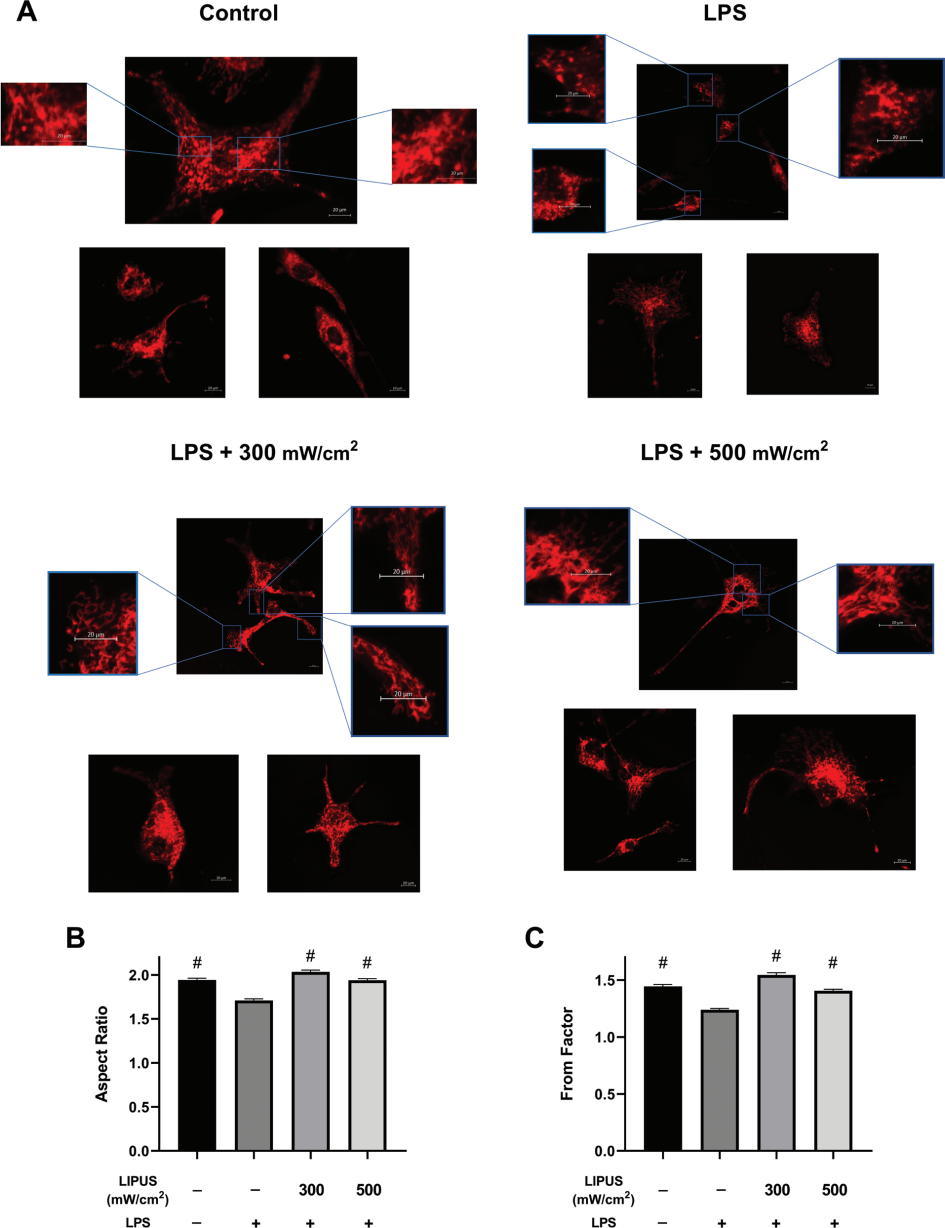
Confocal imaging of mitochondrial morphology and quantitative shape analysis in astrocytes. (A) Representative confocal images (20×) illustrating mitochondrial morphology in control, LPS-treated (0.5 µg/mL, 24 h), and LIPUS-treated (300 and 500 mW/cm^2^, 1 MHz, 50% duty cycle, 15 min) groups. Mitochondria were labeled with MitoTracker™ Red FM and imaged using the Zeiss LSM 900 confocal microscope. Enlarged regions of interest (ROI) are shown to highlight mitochondrial network structures and fragmentation. Each panel includes multiple single-cell images to support qualitative interpretation (scale bars: main panel = 20 µm; zoom insets = 20 µm). (B) Aspect Ratio (AR) and (C) Form Factor (FF) were quantified using the Mitochondria Analyzer plugin in FIJI/ImageJ. For each group, ~100 cells were analyzed, and approximately 20 mitochondria per cell were selected, resulting in a total of ~2000 individual mitochondria per group. Data are shown as mean ± SEM. ^#^*P* < 0.05 vs. LPS group. LIPUS: Low-intensity pulsed ultrasound; LPS: lipopolysaccharide.

Consistent with the molecular findings, confocal microscopy analysis revealed substantial alterations in mitochondrial morphology following LPS exposure and their reversal upon LIPUS treatment. In the LPS group, mitochondria appeared shorter, more rounded, and highly fragmented, as visualized in representative confocal images (Figure 3A and Supplementary Figure 1). Quantitative morphometric analysis showed a significant reduction in both the AR and FF values, indicating disrupted mitochondrial elongation and branching (Figure 3B, C). Notably, LIPUS exposure at 300 and 500 mW/cm^2^ restored mitochondrial structure, as confirmed by improved AR and FF metrics compared to cells subjected to LPS-induced stress. These results suggest that LIPUS application preserves mitochondrial network integrity and attenuates LPS-induced structural fragmentation in astrocytes.

### LIPUS treatment attenuated the mitochondrial unfolded protein response and PGC1α mRNA expression induced by LPS

**Figure 4:**
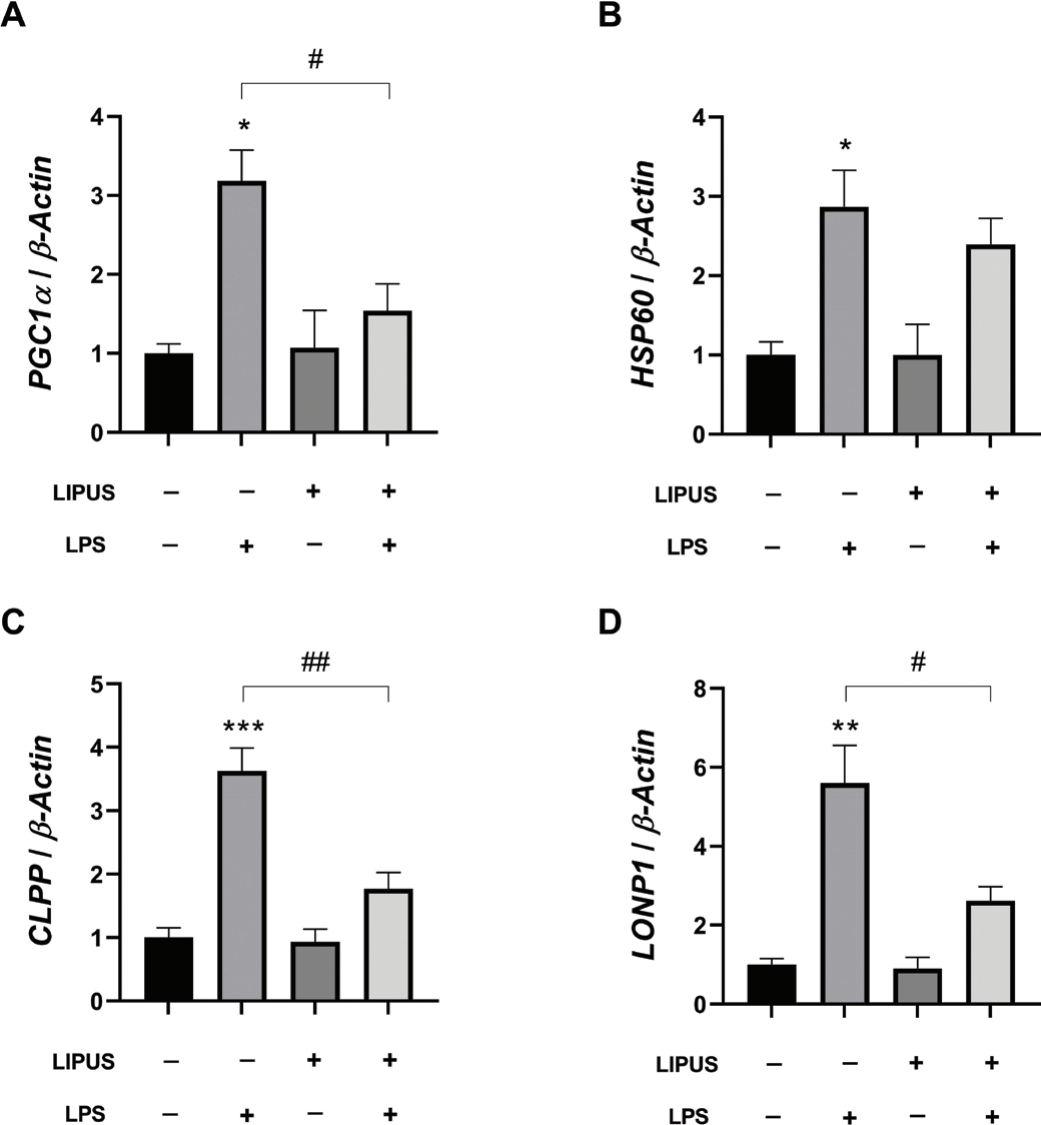
Effect of LIPUS on mRNA levels of mitochondrial protein folding and biogenesis-related genes. mRNA expressions of *PGC1α* (A), *HSP60* (B), *CLPP* (C), and *LONP1* (D) in LPS-stimulated (0.5 µg/mL for 24 h) astrocytes following LIPUS (1 MHz, 50% duty cycle, 300 mW/cm^2^ for 15 min.) treatment after 4h. Control cells were treated with endotoxin-free water. Fold changes were normalized to *β-actin* and calculated relative to control. Data are presented as mean ± SEM. qPCR reactions were run in triplicates in three independent experiments. ***P < 0.001, **P < 0.01, *P < 0.05 vs control and ^##^*P* < 0.01, ^#^*P* < 0.05 vs LPS. LIPUS: Low-intensity pulsed ultrasound; LPS: lipopolysaccharide.

Peroxisome proliferator-activated receptor-gamma coactivator α (PGC1α), a key protein in mitochondrial biogenesis, respiration, and inflammation, plays a role in maintaining mitochondrial dynamics and regulating upstream mechanisms of mitochondrial protein quality control (35). The effect of LIPUS treatment on the mRNA expression levels of *PGC1α* and genes involved in the mitochondrial unfolded protein response (mtUPR) in LPS-stimulated astrocytes is shown in Figure 4. LPS application caused a significant increase in *PGC1α* expression. LIPUS treatment was effective in attenuating this increase (Figure 4A). LIPUS treatment also significantly reduced the mRNA expression levels of ATP-dependent Clp protease proteolytic subunit (*CLPP*) and Lon Peptidase 1 (*LONP1*) genes compared to LPS-treated cells (Figure 4C and D). However, the decrease in mRNA expression levels of the mitochondrial chaperone heat shock protein 60 (*HSP60*) was not statistically significant (Figure 4B).

### LIPUS therapy decreased the mRNA levels of mitochondrial antioxidant enzymes while limiting the LPS-induced rise in ROS

**Figure 5:**
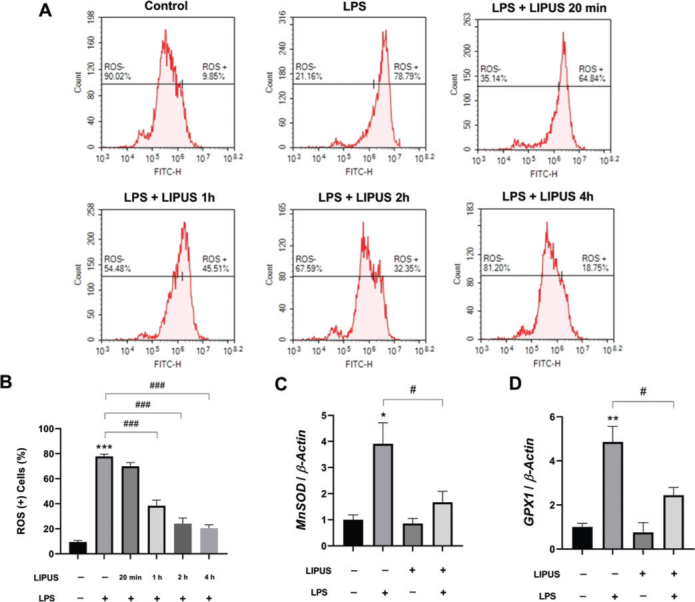
Effect of LIPUS on reactive oxygen species (ROS) profiles and mitochondrial antioxidant enzyme mRNA levels. Quantitative analysis of intracellular ROS levels in LPS-stimulated (0.5 µg/mL for 24 h) astrocytes following LIPUS treatment (1 MHz, 50% duty cycle, 300 mW/cm^2^ for 15 min) using a DCFDA-based flow cytometry assay. Representative flow cytometry histograms showing ROS-negative and ROS-positive cell populations at different time points after LIPUS application (20 min, 1 h, 2 h, and 4 h) are presented (A). The bar graph summarizes the percentage of ROS-positive cells across experimental groups (B). mRNA expressions of *MnSOD* (C), and *GPX1* (D) in LPS-stimulated (0.5 µg/mL for 24 h) astrocytes following LIPUS (1 MHz, 50% duty cycle, 300 mW/cm^2^ for 15 min.) treatment after 4h. Control cells were treated with endotoxin-free water. Data are presented as mean ± SEM. qPCR reactions were run in triplicates in three independent experiments. Fold changes were normalized to *β-actin* and calculated relative to control. ***P < 0.001, **P < 0.01, *P < 0.05 vs control and ^###^*P* < 0.001, ^#^*P* < 0.05 vs. LPS. LIPUS: Low-intensity pulsed ultrasound; LPS: lipopolysaccharide; DCFDA: 2′,7′-dichlorodihydrofluorescein diacetate.

To evaluate the effect of LIPUS treatment on LPS-induced oxidative stress, intracellular ROS levels were quantified by flow cytometry using the DCFDA assay (Figure 5A). LPS stimulation resulted in a marked increase in the percentage of ROS-positive astrocytes compared to control cells. In contrast, LIPUS treatment significantly reduced LPS-induced ROS accumulation in a time-dependent manner, with a progressive decrease observed at 1, 2, and 4 h following ultrasound application (Figure 5B). In addition, LPS caused a significant increase in the mRNA levels of manganese superoxide dismutase (*MnSOD*) and glutathione peroxidase 1 (*GPX1*) genes expressed in astrocytes. LIPUS treatment limited the mRNA expression of these mRNA levels (Figure 5C and D).

### LIPUS treatment restored LPS-induced mitochondrial membrane potential loss

**Figure 6:**
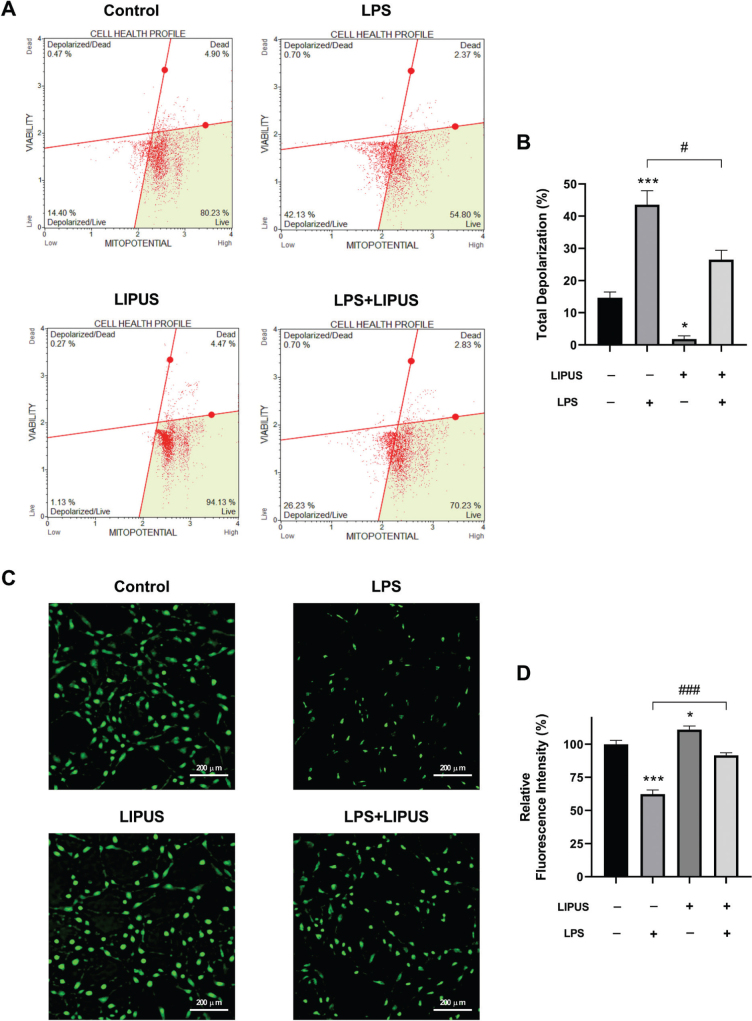
Effect of LIPUS on mitochondrial membrane potential. Quantitative measurement of mitochondrial membrane depolarization in LPS-stimulated (0.5 µg/mL for 24 h) astrocytes following LIPUS (1 MHz, 50% duty cycle, 300 mW/cm^2^ for 15 min.) treatment after 4h. (A). Cell percentages of stained/unstained with MitoPotential dye (polarized/depolarized) and 7-aminoactinomycin D (dead/live) are presented, respectively. The bar graph represents the ratio of total depolarized astrocytes (B). Fluorescence microscopy images of rhodamine 123 staining in experimental groups (C). Fluorescence intensity was presented as a percentage and relative to the control (D). Control cells were treated with endotoxin-free water. Scale bars were indicated in the lower right corner of each image. Data are presented as mean ± SEM of three independent experiments. ***P < 0.001, *P < 0.05 vs control and ^###^*P* < 0.001, ^#^*P* < 0.05, vs. LPS. LIPUS: Low-intensity pulsed ultrasound; LPS: lipopolysaccharide.

The results of the experiment are presented in Figure 6A, which shows the percentage of cells with polarized and depolarized membrane potential. The data revealed that LPS treatment led to a significant increase in the percentage of cells with depolarized membrane potential in comparison to control cells. However, the application of LIPUS alone resulted in a significant reduction in the percentage of cells with depolarized membrane potential. Additionally, LIPUS treatment was able to prevent the loss of membrane potential in cells that were treated with LPS, as demonstrated in Figure 6B. Similar results were obtained with fluorescence microscopy analysis using Rhodamine 123 staining (Figure 6C). Our findings showed that the fluorescence intensity in cells treated with LPS significantly increased compared to control cells. LIPUS treatment increased the decreased membrane fluorescence intensity induced by LPS. Additionally, LIPUS treatment alone showed increased fluorescence compared to control cells (Figure 6D). To further investigate the early effects of LIPUS on mitochondrial membrane potential, Rhodamine 123–based flow cytometry analysis was performed at earlier time points following LIPUS application. As shown in Supplementary Figure 2, LIPUS treatment progressively reduced the percentage of depolarized cells as early as 20 min after application, with further improvements observed at 1 h and 2 h. These findings indicate that LIPUS rapidly initiates mitochondrial functional recovery prior to the later time points analyzed in the main experiments.

## Discussion

Microglia and astroglia in the brain are both considered innate immune cells that contribute to the development of inflammation in various brain diseases (36). These cells release inflammatory mediators such as ROS, interleukins, chemokines, and prostanoids, which can further exacerbate the inflammatory response. Astrocytes generally exhibit a slower and sustained response to inflammatory stimuli, while microglia respond rapidly and acutely. This difference in response kinetics may contribute to the timing and magnitude of their inflammatory actions (37). On the one hand, microglia play a crucial role in clearing cellular debris and pathogens, promoting tissue repair, and supporting neuronal survival. These mechanisms are essential for maintaining brain homeostasis and promoting recovery after injuries or infections. On the other hand, excessive or chronic activation of microglia may lead to sustained neuroinflammation, increased oxidative stress, and subsequent neuronal damage. (38). Overall, both microglia and astroglia play important roles in neuroinflammation and contribute to the pathogenesis of brain diseases (38, 39). Mitochondrial dysfunction and neuroinflammation are two major pathological characteristics that are strongly linked to the development of neurodegenerative diseases (40, 41). These pathological features are closely associated with the development of hallmark features of these diseases and have been identified as interdependent pathological factors (42). This suggests that mitochondrial dysfunction and neuroinflammation may work together to contribute to the progression of neurodegenerative diseases.

**Figure 7:**
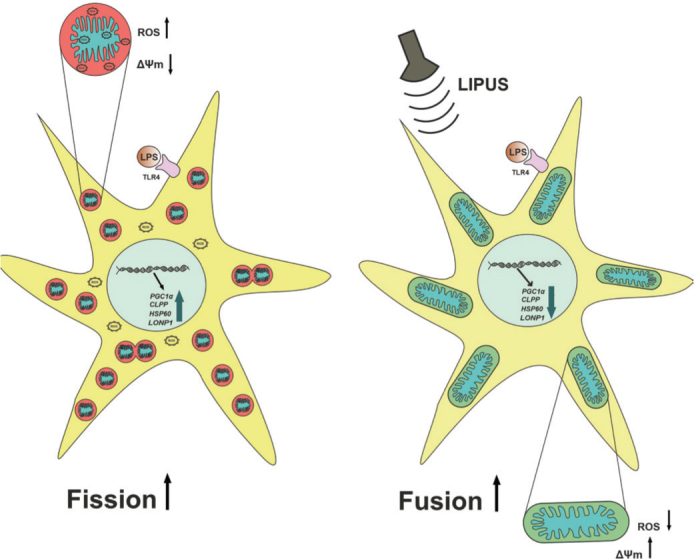
Schematic illustration of the proposed mechanism by which LIPUS alleviates LPS-induced mitochondrial dysfunction in astrocytes. (Left) Upon lipopolysaccharide (LPS) stimulation, astrocytes exhibit increased mitochondrial fragmentation (fission), elevated reactive oxygen species (ROS) levels, and a reduction in mitochondrial membrane potential (ΔΨm). These alterations are accompanied by accompanied by increased mRNA expression, reflecting mitochondrial stress response activation. (Right) In contrast, LIPUS application promotes mitochondrial fusion, reduces ROS levels, and restores ΔΨm. In addition, LIPUS prevents the LPS-induced downregulation of *PGC1α, CLPP, HSP60*, and *LONP1*, suggesting a restoration of mitochondrial homeostasis through both structural and transcriptional regulation. LIPUS: Low-intensity pulsed ultrasound.

Studies investigating the protective roles of LIPUS have shown that LIPUS can prevent cellular damage by activating various mechanisms in cells at specific power densities (5, 43). These mechanisms include inhibiting inflammation (5), and suppressing apoptotic signals (43), and activating mitophagy (44). In this study, we demonstrated for the first time the effects of LIPUS application on mitochondrial dynamics in astrocytes. In addition, we identified the role of LIPUS in alleviating LPS-induced mitochondrial dysfunction (Figure 7).

The mitochondria found in astrocytes are crucial for maintaining brain energy metabolism and regulating the immune system in the brain (45). When there is inflammation in the CNS, astrocytes respond in different ways depending on the location of the damage. In particular, the mitochondria within astrocytes are affected. During neuroinflammation and tissue injury, the balance between the processes of mitochondrial fusion and fission is disrupted in astrocytes. This faulty regulation of mitochondrial dynamics has been linked to the process of astrogliosis, which is the activation and proliferation of astrocytes in response to injury (46). In cases of severe brain damage and inflammation, astrocytic mitochondria are prone to fragmentation through a process known as fission. This is accompanied by an increase in the expression of a protein called phosphorylated-DRP1 (Ser616), which causes the mitochondria to fragment even further (47). Excessive mitochondrial fission and fragmentation have been linked to neuroinflammation and the development of degenerative diseases. Joshi et al. have reported on the role of mitochondrial fragmentation in the context of astrocytic inflammatory activation and neurodegenerative diseases (48). Additionally, a study has shown that overexpression of the fusion protein MFN2 under LPS-induced neuroinflammation conditions prevented microglial activation and cytokine release in mice, leading to the improvement of behavioral disorders. It has been suggested that this protein plays a role in the mechanistic link between mitochondrial dysfunction and neuroinflammation by suppressing the mitochondrial fragmentation triggered by LPS (17).

The LIPUS therapy on mitochondrial dynamics are still poorly understood and very few studies have investigated this relationship. However, a recent study has shed some light on this topic by demonstrating that LIPUS, at intensities of 100 or 200 mW/cm^2^, can suppress the mitochondrial fragmentation induced by advanced glycation end products and promote a shift toward fusion dynamics (49). These findings provide valuable insights into the potential use of LIPUS as a therapeutic strategy for mitigating mitochondrial dysfunction in pathological conditions associated with mitochondrial fragmentation and impaired fusion–fission dynamics. This study demonstrates that the effects of LIPUS on astrocytes are dependent on both the applied power intensity and the duration of exposure. Considering that ultrasound waves exert mechanical stimulation on biological tissues, suboptimal intensities may fail to elicit sufficient cellular responses, whereas excessively high intensities may trigger unwanted stress reactions. While appropriately tuned ultrasound exposure exerts anti-inflammatory and cytoprotective effects, overly intense stimulation may be ineffective or even detrimental (50). Therefore, careful optimization of LIPUS parameters is critical for therapeutic efficacy. Regarding the temporal aspect, repeated or prolonged LIPUS application appears to enhance its protective effects. During the acute phase of inflammation, short-term exposure may yield transient improvements, whereas extended treatment might support more durable cellular adaptations involving antioxidant defense, mitophagy, and mitochondrial biogenesis. Indeed, in the study by Chen et al., LIPUS was administered daily for seven consecutive days to suppress neuroinflammation and improve cognitive function (51). Importantly, our early time-point analysis revealed that LIPUS rapidly initiates transcriptional regulation of mitochondrial dynamics, as evidenced by progressive upregulation of fusion-related genes and concomitant suppression of fission markers within 30 min to 2 h after stimulation. These findings indicate that LIPUS-induced modulation of mitochondrial dynamics begins at an early phase, preceding the maximal mRNA-level changes observed at later time points. In our model, the optimal effect observed at 300 mW/cm^2^ aligns with previous literature reporting that moderate intensities of LIPUS are most effective in eliciting biological responses. Additionally, it is known that the cellular effects of LIPUS are transient, peaking within a few hours and then diminishing. For example, a study in osteoblastic cells showed that a single LIPUS session led to a peak expression of early response genes around 3 h, followed by a return to baseline levels within 6–12 h (52). Therefore, our finding that mitochondrial fusion mRNAs peaked at 4 h and declined thereafter is consistent with a rapid but transient mechanotransduction response. The mRNA-level findings clearly indicate that LIPUS induces fluctuations in gene expression in a time-dependent manner. These considerations highlight the importance of optimizing both the intensity and duration of LIPUS application to achieve maximal efficacy. Our findings support the notion that, under optimal conditions, LIPUS may exert a potent mitochondrial regulatory effect in astrocytic inflammatory responses.

The significance of maintaining mitochondrial homeostasis in relation to neuroinflammatory responses has gained increasing attention. Mitochondrial dysfunction can lead to the production of ROS and accumulation of misfolded and unfolded proteins, ultimately resulting in mitochondrial stress responses (53). PGC1α is a critical mediator of both mitochondrial dynamics and inflammation. As a coactivator, PGC1α enhances the transcriptional activity of multiple pathways that regulate inflammation, mitochondrial respiration, and biogenesis (35) as well as mitochondrial protein quality control (54). A recent study has reported that LPS promotes the upregulation of mitochondrial stress proteins CLPP, HSP60, and LONP1 in microglial cells by stimulating cytokine release (55). In another study, it was found that the mRNA expression of *PGC1α* increased following LPS application and that *HSP60*, which is an essential component of mitochondrial protein transport, was upregulated as well in neurons (56). In this study, we observed that 24-h LPS stimulation significantly increased mRNA levels of *PGC1α, CLPP, HSP60*, and *LONP1* genes, which is consistent with previous studies. While LIPUS treatment alone did not induce any changes in the relevant mRNA levels, it was effective in preventing the LPS-induced elevation for *PGC1α, CLPP*, and *LONP1*.

It has previously been demonstrated that LPS induces ROS production in astrocyte cells (57, 58). In addition, the presence of fragmented mitochondria in astrocytes results in an increase in the generation of ROS and a reduction in ATP production. This, in turn, activates the nuclear factor kappa B (NF-κB) pathway and leads to the release of proinflammatory cytokines, thereby initiating a toxic feedback loop of chronic neuroinflammation that can ultimately result in neurotoxicity (59). It has previously been found that ultrasound treatment regulates mitochondrial complex 1 activity and mtROS formation against rotenone-induced oxidative damage (60). It has been demonstrated that LIPUS is capable of scavenging ROS (61) and reduce ROS levels in chondrocytes pre-treated with LPS, in a manner that was both dose-dependent and intensity-dependent (62). This study demonstrated, in line with previous research, that LPS induced a significant increase in ROS levels in astrocytes. However, LIPUS treatment contributed to a reduction in ROS levels, both due to its ability to scavenge ROS and its biochemical and/or mechanical effects on mitochondrial dynamics. This finding was supported by an increase in mitochondrial antioxidant enzyme mRNA levels. The decrease in ROS levels was accompanied by a reduction of enzyme mRNA expression. Importantly, time-course analyses revealed that the reduction in intracellular ROS levels was initiated rapidly following LIPUS application, indicating that LIPUS exerts early regulatory effects on mitochondrial redox homeostasis. Such rapid modulation of ROS may contribute to the interruption of the feed-forward inflammatory signaling cascade at an early stage.

Mitochondrial dysfunction is a condition that causes a decrease in the energy production capacity of the mitochondria. This condition can lead to a disturbance in the electrochemical gradient within the mitochondria, ultimately resulting in a decrease in mitochondrial membrane potential (63). In a study, it was found that pretreatment with LIPUS suppressed mitochondrial membrane depolarization and cytochrome c release in neurons treated with neuronal toxin (64). Our study showed that the impairment in mitochondrial membrane potential due to LPS could be reversed with LIPUS treatment. Furthermore, LIPUS alone was effective in boosting cell energy metabolism by reducing the proportion of cells with depolarized membrane potential compared to control cells. Notably, additional time-course analyses revealed that this protective effect of LIPUS on mitochondrial membrane potential was initiated rapidly, within minutes after application, suggesting an early and direct modulation of mitochondrial function.

Overall, our results indicate that using LIPUS as a therapeutic strategy could be a novel approach to address neuroinflammation, mitochondrial dysfunction, and related diseases. However, further research is necessary to explore how LIPUS functions mechanistically in various types of neurodegenerative disorders. The absence of experiments on microglia cells represents a valid limitation of our research. Conducting such experiments could provide a more comprehensive understanding of the interplay between different glial cell types and their respective responses to LIPUS treatment. In considering the limitations of our study, another notable aspect is the absence of *in vivo* experiments. We acknowledge that the translation of our findings to *in vivo* settings is essential for clinical relevance and to understand the broader implications in a more complex biological context.

## Supplementary Material



## References

[1] Gitler AD, Dhillon P, Shorter J. Neurodegenerative disease: models, mechanisms, and a new hope. Dis Model Mech. 2017;10:499–502. doi: 10.1242/dmm.030205 28468935 PMC5451177

[2] Batista CRA, Gomes GF, Candelario-Jalil E, Fiebich BL, De Oliveira ACP. Lipopolysaccharide-induced neuroinflammation as a bridge to understand neurodegeneration. Int J Mol Sci. 2019;20:2293. doi: 10.3390/ijms20092293 31075861 PMC6539529

[3] Nava Catorce M, Gevorkian G. LPS-induced murine neuroinflammation model: main features and suitability for pre-clinical assessment of nutraceuticals. Curr Neuropharmacol. 2016;14:155–64. doi: 10.2174/1570159X14666151204122017 26639457 PMC4825946

[4] Schwartz M, Butovsky O, Brück W, Hanisch U-K. Microglial phenotype: is the commitment reversible? Trends Neurosci. 2006;29:68–74. doi: 10.1016/j.tins.2005.12.005 16406093

[5] Chang J-W, Wu M-T, Song W-S, Yang F-Y. Ultrasound stimulation suppresses LPS-induced proinflammatory responses by regulating NF-κB and CREB activation in microglial cells. Cereb Cortex. 2020;30:4597–606. doi: 10.1093/cercor/bhaa062 32248223

[6] Farina C, Aloisi F, Meinl E. Astrocytes are active players in cerebral innate immunity. Trends Immunol. 2007;28:138–45. doi: 10.1016/j.it.2007.01.005 17276138

[7] Kolac UK, Ustuner MC, Tekin N, Ustuner D, Colak E, Entok E. The anti-inflammatory and antioxidant effects of Salvia officinalis on lipopolysaccharide-induced inflammation in rats. J Med Food. 2017;20:1193–200. doi: 10.1089/jmf.2017.0035 29131698

[8] Zhang F-X, Xu R-S. Juglanin ameliorates LPS-induced neuroinflammation in animal models of Parkinson’s disease and cell culture via inactivating TLR4/NF-κB pathway. Biomed Pharmacother. 2018;97:1011–19. doi: 10.1016/j.biopha.2017.08.132 29136779

[9] Yang L, Zhou R, Tong Y, Chen P, Shen Y, Miao S, et al. Neuroprotection by dihydrotestosterone in LPS-induced neuroinflammation. Neurobiol Dis. 2020;140:104814. doi: 10.1016/j.nbd.2020.104814 32087283

[10] Lopes PC. LPS and neuroinflammation: a matter of timing. Inflammopharmacology. 2016;24:291–3. doi: 10.1007/s10787-016-0283-2 27645902

[11] Qin L, Wu X, Block ML, Liu Y, Breese GR, Hong JS, et al. Systemic LPS causes chronic neuroinflammation and progressive neurodegeneration. Glia. 2007;55:453–62. doi: 10.1002/glia.20467 17203472 PMC2871685

[12] Chan DC. Fusion and fission: interlinked processes critical for mitochondrial health. Ann Rev Genet. 2012;46:265–87. doi: 10.1146/annurev-genet-110410-132529 22934639

[13] Pernas L, Scorrano L. Mito-morphosis: mitochondrial fusion, fission, and cristae remodeling as key mediators of cellular function. Ann Rev Physiol. 2016;78:505–31. doi: 10.1146/annurev-physiol-021115-105011 26667075

[14] Burté F, Carelli V, Chinnery PF, Yu-Wai-Man P. Disturbed mitochondrial dynamics and neurodegenerative disorders. Nat Rev Neurol. 2015;11:11–24. doi: 10.1038/nrneurol.2014.228 25486875

[15] Senft D, Ze’ev AR. Regulators of mitochondrial dynamics in cancer. Curr Opin Cell Biol. 2016;39:43–52. doi: 10.1016/j.ceb.2016.02.001 26896558 PMC4828329

[16] Kolac UK, Donmez Yalcin G, Yalcin A. Chemical inhibition of mitochondrial fission improves insulin signaling and subdues hyperglycemia induced stress in placental trophoblast cells. Mol Biol Rep. 2022;50:493–506. doi: 10.1007/s11033-022-07959-0 36352179

[17] Harland M, Torres S, Liu J, Wang X. Neuronal mitochondria modulation of LPS-induced neuroinflammation. J Neurosci. 2020;40:1756–65. doi: 10.1523/JNEUROSCI.2324-19.2020 31937559 PMC7046320

[18] Stavropoulos F, Sargiannidou I, Potamiti L, Kagiava A, Panayiotidis MI, Bae JH, et al. Aberrant mitochondrial dynamics and exacerbated response to neuroinflammation in a novel mouse model of CMT2A. Int J Mol Sci. 2021;22:11569. doi: 10.3390/ijms222111569 34769001 PMC8584238

[19] Ma B, Yu J, Xie C, Sun L, Lin S, Ding J, et al. Toll-like receptors promote mitochondrial translocation of nuclear transcription factor nuclear factor of activated T-cells in prolonged microglial activation. J Neurosci. 2015;35:10799–814. doi: 10.1523/JNEUROSCI.2455-14.2015 26224862 PMC4518054

[20] Sen D. DOR agonist (SNC-80) exhibits anti-parkinsonian effect via downregulating UPR/oxidative stress signals and inflammatory response in vivo. Neurosci Lett. 2018;678:29–36. doi: 10.1016/j.neulet.2018.04.055 29727730

[21] Urrutia PJ, Mena NP, Núñez MT. The interplay between iron accumulation, mitochondrial dysfunction, and inflammation during the execution step of neurodegenerative disorders. Front Pharmacol. 2014;5:38. doi: 10.3389/fphar.2014.00038 24653700 PMC3948003

[22] Sun J, Song F-H, Wu J-Y, Zhang L-Q, Li D-Y, Gao S-J, et al. Sestrin2 overexpression attenuates osteoarthritis pain via induction of AMPK/PGC-1α-mediated mitochondrial biogenesis and suppression of neuroinflammation. Brain Behav Immun. 2022;102:53–70. doi: 10.1016/j.bbi.2022.02.015 35151829

[23] Leighton TG. What is ultrasound? Prog Biophys Mol Biol. 2007;93:3–83. doi: 10.1016/j.pbiomolbio.2006.07.026 17045633

[24] Jiang X, Savchenko O, Li Y, Qi S, Yang T, Zhang W, et al. A review of low-intensity pulsed ultrasound for therapeutic applications. IEEE Trans Biomed Eng. 2018;66:2704–18. doi: 10.1109/TBME.2018.2889669 30596564

[25] Ning GZ, Song WY, Xu H, Zhu RS, Wu QL, Wu Y, et al. Bone marrow mesenchymal stem cells stimulated with low-intensity pulsed ultrasound: better choice of transplantation treatment for spinal cord injury: treatment for SCI by LIPUS-BMSCs transplantation. CNS Neurosci Ther. 2019;25:496–508. doi: 10.1111/cns.13071 30294904 PMC6488928

[26] Wu C-T, Yang T-H, Chen M-C, Chung Y-P, Guan S-S, Long L-H, et al. Low intensity pulsed ultrasound prevents recurrent ischemic stroke in a cerebral ischemia/reperfusion injury mouse model via brain-derived neurotrophic factor induction. Int J Mol Sci. 2019;20:5169. doi: 10.3390/ijms20205169 31635269 PMC6834125

[27] Song W-S, Sung C-Y, Ke C-H, Yang F-Y. Anti-inflammatory and neuroprotective effects of transcranial ultrasound stimulation on Parkinson’s disease. Ultrasound Med Biol. 2022;48:265–74. doi: 10.1016/j.ultrasmedbio.2021.10.001 34740497

[28] Nakao J, Fujii Y, Kusuyama J, Bandow K, Kakimoto K, Ohnishi T, et al. Low-intensity pulsed ultrasound (LIPUS) inhibits LPS-induced inflammatory responses of osteoblasts through TLR4–MyD88 dissociation. Bone. 2014;58:17–25. doi: 10.1016/j.bone.2013.09.018 24091132

[29] Xu M, Wang L, Wu S, Dong Y, Chen X, Wang S, et al. Review on experimental study and clinical application of low-intensity pulsed ultrasound in inflammation. Quant Imaging Med Surg. 2021;11:443. doi: 10.21037/qims-20-680 33392043 PMC7719927

[30] Liu S, Zhou M, Li J, Hu B, Jiang D, Huang H, et al. LIPUS inhibited the expression of inflammatory factors and promoted the osteogenic differentiation capacity of hPDLCs by inhibiting the NF-κB signaling pathway. J Periodontal Res. 2020;55:125–40. doi: 10.1111/jre.12696 31541455

[31] da Silva Junior EM, Mesquita-Ferrari RA, Franca CM, Andreo L, Bussadori SK, Fernandes KPS. Modulating effect of low intensity pulsed ultrasound on the phenotype of inflammatory cells. Biomed Pharmacother. 2017;96:1147–53. doi: 10.1016/j.biopha.2017.11.108 29191696

[32] Li X, Zhong Y, Zhou W, Song Y, Li W, Jin Q, et al. Low-intensity pulsed ultrasound (LIPUS) enhances the anti-inflammatory effects of bone marrow mesenchymal stem cells (BMSCs)-derived extracellular vesicles. Cell Mol Biol Lett. 2023;28:9. doi: 10.1186/s11658-023-00422-3 36717768 PMC9885645

[33] Cui W, Li H, Guan R, Li M, Yang B, Xu Z, et al. Efficacy and safety of novel low-intensity pulsed ultrasound (LIPUS) in treating mild to moderate erectile dysfunction: a multicenter, randomized, double-blind, sham-controlled clinical study. Transl Androl Urol. 2019;8:307. doi: 10.21037/tau.2019.07.03 31555554 PMC6732092

[34] Turkkol A, Kolac UK, Donmez Yalcin G, Bilgin MD, Yalcin A, Bilgen M. Enhancing sonodynamic therapy in prostate cancer: cavitation-induced cytotoxicity and mitochondrial unfolded protein response disruption. Cell Biochem Biophys. 2025;83:3353–67. doi: 10.1007/s12013-025-01717-2 40131613

[35] Botta A, Laher I, Beam J, DeCoffe D, Brown K. Short term exercise induces PGC-1o. ameliorates inflammation and increases mitochondrial. PLoS One. 2013;8:e70248. doi: 10.1371/journal.pone.0070248 23936397 PMC3731348

[36] Tzeng S-F, Hsiao H-Y, Mak O-T. Prostaglandins and cyclooxygenases in glial cells during brain inflammation. Curr Drug Targets Inflamm Allergy. 2005;4:335–40. doi: 10.2174/1568010054022051 16101543

[37] Matejuk A, Ransohoff RM. Crosstalk between astrocytes and microglia: an overview. Front Immunol. 2020;11:1416. doi: 10.3389/fimmu.2020.01416 32765501 PMC7378357

[38] Gebicke-Haerter PJ. Microglia in neurodegeneration: molecular aspects. Microsc Res Tech. 2001;54:47–58. doi: 10.1002/jemt.1120 11526957

[39] Kipp M, Karakaya S, Johann S, Kampmann E, Mey J, Beyer C. Oestrogen and progesterone reduce lipopolysaccharide-induced expression of tumour necrosis factor-α and interleukin-18 in midbrain astrocytes. J Neuroendocrinol. 2007;19:819–22. doi: 10.1111/j.1365-2826.2007.01588.x 17850464

[40] Heneka MT, Carson MJ, El Khoury J, Landreth GE, Brosseron F, Feinstein DL, et al. Neuroinflammation in Alzheimer’s disease. Lancet Neurol. 2015;14:388–405. doi: 10.1016/S1474-4422(15)70016-5 25792098 PMC5909703

[41] Gao J, Wang L, Liu J, Xie F, Su B, Wang X. Abnormalities of mitochondrial dynamics in neurodegenerative diseases. Antioxidants. 2017;6:25. doi: 10.3390/antiox6020025 28379197 PMC5488005

[42] Wilkins HM, Swerdlow RH. Relationships between mitochondria and neuroinflammation: implications for Alzheimer’s disease. Curr Top Med Chem. 2016;16:849–57. doi: 10.2174/1568026615666150827095102 26311426 PMC5480219

[43] Zhou J-X, Liu Y-J, Chen X, Zhang X, Xu J, Yang K, et al. Low-intensity pulsed ultrasound protects retinal ganglion cell from optic nerve injury induced apoptosis via yes associated protein. Front Cell Neurosci. 2018;12:160. doi: 10.3389/fncel.2018.00160 29950973 PMC6008403

[44] Chiang P-K, Yang F-Y. A potential treatment of low intensity pulsed ultrasound on cavernous nerve injury for erectile dysfunction. Med Hypotheses. 2019;122:19–21. doi: 10.1016/j.mehy.2018.10.014 30593410

[45] Bélanger M, Allaman I, Magistretti PJ. Brain energy metabolism: focus on astrocyte-neuron metabolic cooperation. Cell Metab. 2011;14:724–38. doi: 10.1016/j.cmet.2011.08.016 22152301

[46] Motori E, Puyal J, Toni N, Ghanem A, Angeloni C, Malaguti M, et al. Inflammation-induced alteration of astrocyte mitochondrial dynamics requires autophagy for mitochondrial network maintenance. Cell Metab. 2013;18:844–59. doi: 10.1016/j.cmet.2013.11.005 24315370

[47] Sheng Z-H. Mitochondrial trafficking and anchoring in neurons: new insight and implications. J Cell Biol. 2014;204:1087–98. doi: 10.1083/jcb.201312123 24687278 PMC3971748

[48] Joshi AU, Minhas PS, Liddelow SA, Haileselassie B, Andreasson KI, Dorn GW, et al. Fragmented mitochondria released from microglia trigger A1 astrocytic response and propagate inflammatory neurodegeneration. Nat Neurosci. 2019;22:1635–48. doi: 10.1038/s41593-019-0486-0 31551592 PMC6764589

[49] Chen Y, Xiao M, Zhao L, Huang Y, Lin Y, Xie T, et al. Low-intensity pulsed ultrasound counteracts advanced glycation end products-induced corpus cavernosal endothelial cell dysfunction via activating mitophagy. Int J Mol Sci. 2022;23:14887. doi: 10.3390/ijms232314887 36499213 PMC9740783

[50] Cao Q, Liu L, Hu Y, Cao S, Tan T, Huang X, et al. Low-intensity pulsed ultrasound of different intensities differently affects myocardial ischemia/reperfusion injury by modulating cardiac oxidative stress and inflammatory reaction. Front Immunol. 2023;14:1248056. doi: 10.3389/fimmu.2023.1248056 37744362 PMC10513435

[51] Chen T-T, Lan T-H, Yang F-Y. Low-intensity pulsed ultrasound attenuates LPS-induced neuroinflammation and memory impairment by modulation of TLR4/NF-κB signaling and CREB/BDNF expression. Cereb Cortex. 2019;29:1430–8. doi: 10.1093/cercor/bhy039 30873554

[52] Sena K, Leven RM, Mazhar K, Sumner DR, Virdi AS. Early gene response to low-intensity pulsed ultrasound in rat osteoblastic cells. Ultrasound Med Biol. 2005;31:703–8. doi: 10.1016/j.ultrasmedbio.2005.01.013 15866420

[53] Wang S, Zheng L, Zhao T, Zhang Q, Liu Y, Sun B, et al. Inhibitory effects of walnut (Juglans regia) peptides on neuroinflammation and oxidative stress in lipopolysaccharide-induced cognitive impairment mice. J Agr Food Chem. 2020;68:2381–92. doi: 10.1021/acs.jafc.9b07670 32037817

[54] Zhang Q, Lei Y-H, Zhou J-P, Hou Y-Y, Wan Z, Wang H-L, et al. Role of PGC-1α in mitochondrial quality control in neurodegenerative diseases. Neurochem Res. 2019;44:2031–43. doi: 10.1007/s11064-019-02858-6 31410709

[55] Zhu J, Lee MJ, An JH, Oh E, Chung W, Heo JY. ATF5 attenuates the secretion of pro-inflammatory cytokines in activated microglia. Int J Mol Sci. 2023;24:3322. doi: 10.3390/ijms24043322 36834738 PMC9961550

[56] Anne Stetler R, Leak RK, Yin W, Zhang L, Wang S, Gao Y, et al. Mitochondrial biogenesis contributes to ischemic neuroprotection afforded by LPS pre-conditioning. J Neurochem. 2012;123:125–37. doi: 10.1111/j.1471-4159.2012.07951.x 23050650 PMC3623612

[57] Yang C-C, Hsiao L-D, Tseng H-C, Kuo C-M, Yang C-M. Pristimerin inhibits MMP-9 expression and cell migration through attenuating NOX/ROS-dependent NF-κB activation in rat brain astrocytes challenged with LPS. J Inflamm Res. 2020;13:325–41. doi: 10.2147/JIR.S252659 32765041 PMC7381777

[58] Wang Y, Zhao C-S. Sigma-1 receptor activation ameliorates LPS-induced NO production and ROS formation through the Nrf2/HO-1 signaling pathway in cultured astrocytes. Neurosci Lett. 2019;711:134387. doi: 10.1016/j.neulet.2019.134387 31330223

[59] Kaur D, Sharma V, Deshmukh R. Activation of microglia and astrocytes: a roadway to neuroinflammation and Alzheimer’s disease. Inflammopharmacology. 2019;27:663–77. doi: 10.1007/s10787-019-00580-x 30874945

[60] Hada B, Karmacharya MB, Park SR, Choi BH. Low-intensity ultrasound (LIUS) differentially modulates mitochondrial reactive oxygen species (mtROS) generation by three different chemicals in PC12 cells. Free Radic Res. 2021;55:1037–47. doi: 10.1080/10715762.2021.2010730 34814783

[61] Li J, Zhang Q, Ren C, Wu X, Zhang Y, Bai X, et al. Low-intensity pulsed ultrasound prevents the oxidative stress induced endothelial-mesenchymal transition in human aortic endothelial cells. Cell Physiol Biochem. 2018;45:1350–65. doi: 10.1159/000487561 29462805

[62] Zuo D, Tan B, Jia G, Wu D, Yu L, Jia L. A treatment combined prussian blue nanoparticles with low-intensity pulsed ultrasound alleviates cartilage damage in knee osteoarthritis by initiating PI3K/Akt/mTOR pathway. Am J Transl Res. 2021;13:3987. doi: 10.21203/rs.3.rs-77010/v1 34149994 PMC8205753

[63] Hwang HS, Kim HA. Chondrocyte apoptosis in the pathogenesis of osteoarthritis. Int J Mol Sci. 2015;16:26035–54. doi: 10.3390/ijms161125943 26528972 PMC4661802

[64] Zhao L, Feng Y, Shi A, Zhang L, Guo S, Wan M. Neuroprotective effect of low-intensity pulsed ultrasound against MPP+-induced neurotoxicity in PC12 cells: involvement of K2P channels and stretch-activated ion channels. Ultrasound Med Biol. 2017;43:1986–99. doi: 10.1016/j.ultrasmedbio.2017.04.020 28583325

