# Supplementary material for: Low-intensity pulsed ultrasound restores mitochondrial dynamics and function in lipopolysaccharide-stimulated astrocytes
Source: Ups J Med Sci. 2026 Mar 9;131:10.48101/ujms.v131.13678. doi: 10.48101/ujms.v131.13678 (PMC13054932; doi:10.48101/ujms.v131.13678)
Supplement: Supplementary file 1 [file UJMS-131-13678-s1.pdf]

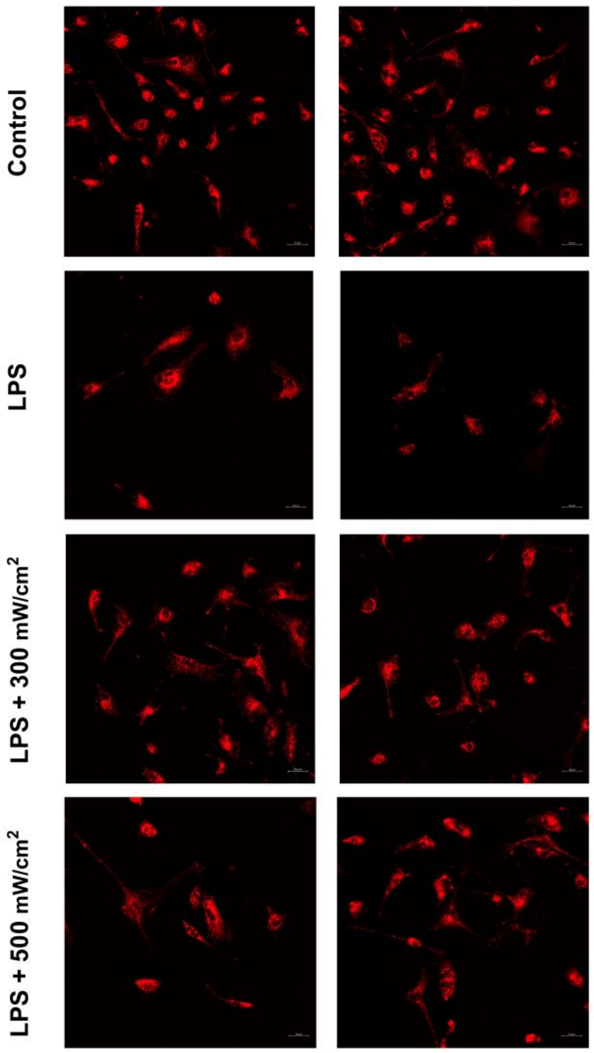

Supplementary Fig. 1 Low-magnification confocal imaging of mitochondrial morphology in astrocytes.

Representative 10× confocal images showing mitochondrial staining in astrocytes under control, LPS-treated (0.5 µg/ml, 24 h), and LIPUS-treated (300 and 500 mW/cm², 1 MHz, 50% duty cycle, 15 min) conditions. Mitochondria were labeled with MitoTracker™ Red FM and imaged using confocal microscope. These wide-field views provide an overview of mitochondrial distribution and cell density across different treatment groups (scale bar: 50 µm).

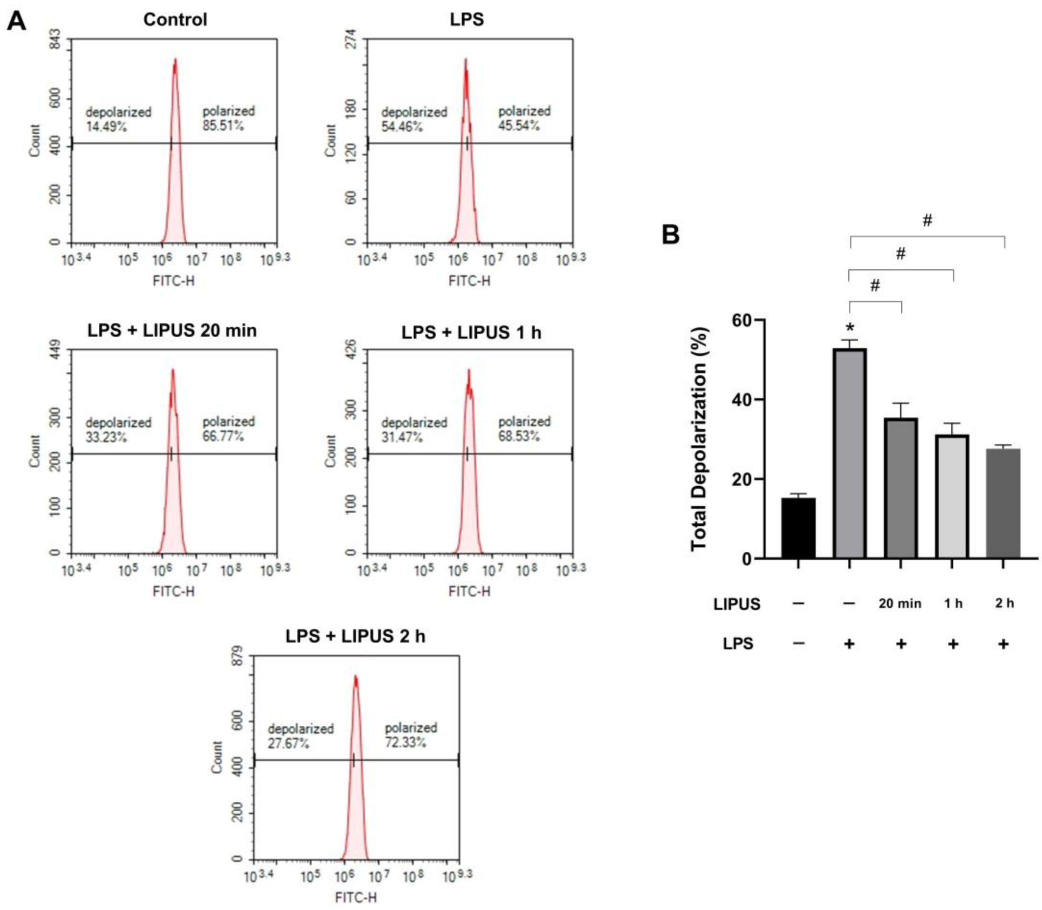

Supplementary Fig. 2 Early effects of LIPUS on mitochondrial membrane potential assessed by Rhodamine 123 flow cytometry.

Representative flow cytometry histograms showing mitochondrial membrane potential (ΔΨm) profiles in control, LPS-treated (0.5 µg/ml for 24 h), and LPS-stimulated astrocytes following LIPUS application at early time points (20 min, 1 h, and 2 h) are presented (A). Mitochondrial membrane depolarization was assessed using Rhodamine 123 staining, and polarized and depolarized cell populations were identified based on fluorescence intensity. The bar graph summarizes the percentage of total depolarized cells across experimental groups (B). Flow cytometric analysis was performed using the FITC channel with 20,000 events acquired per sample, and identical gating parameters were applied to all groups. Data are presented as mean ± SEM of three independent experiments. *$P < 0.05$ vs control and $^{\#}P < 0.05$ vs LPS.

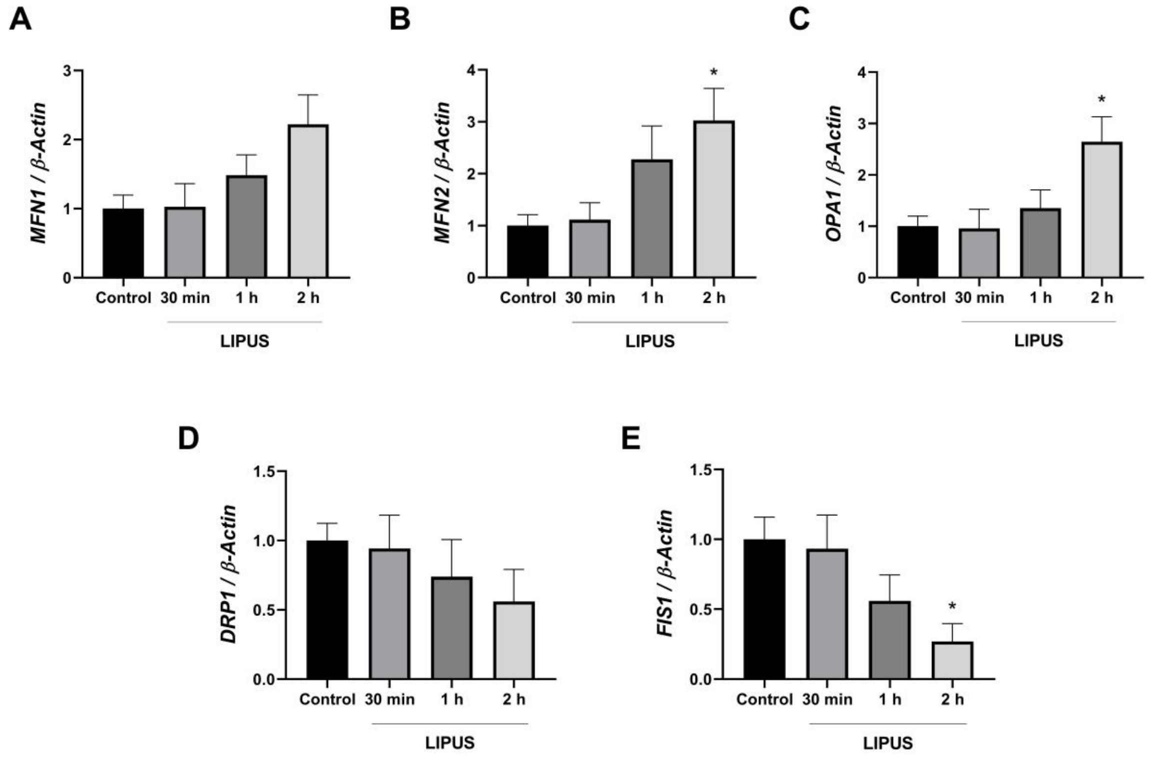

Supplement Fig. 3 Time-dependent effects of LIPUS on mitochondrial fission and fusion gene expression at early time points.

mRNA expression levels of mitochondrial fusion genes *MFN1* (A), *MFN*2 (B), and *OPA1* (C), and fission-related genes *DRP1* (D) and *FIS1* (E) were analyzed at 30 min, 1 h, and 2 h following LIPUS treatment. Fold changes were normalized to *β-actin* and calculated relative to control cells. Data are presented as mean ± SEM. qPCR analysis was performed in triplicates. *$P < 0.05$ vs control.
